# Role of Macrophage Migration Inhibitory Factor (MIF) in Pollen-Induced Allergic Conjunctivitis and Pollen Dermatitis in Mice

**DOI:** 10.1371/journal.pone.0115593

**Published:** 2015-02-03

**Authors:** Yuka Nagata, Yoko Yoshihisa, Kenji Matsunaga, Mati Ur Rehman, Nobuyuki Kitaichi, Tadamichi Shimizu

**Affiliations:** 1 Department of Dermatology, Graduate School of Medicine and Pharmaceutical Sciences, University of Toyama, Toyama, Japan; 2 Department of Ophthalmology, Health Sciences University of Hokkaido, Sapporo, Japan; McGill University, CANADA

## Abstract

Pollen is a clinically important airborne allergen and one of the major causes of allergic conjunctivitis. A subpopulation of patients with atopic dermatitis (AD) are also known to have exacerbated skin eruptions on the face, especially around the eyelids, after contact with pollen. This pollen-induced skin reaction is now known as pollen dermatitis. Macrophage migration inhibitory factor (MIF) is a pluripotent cytokine that plays an essential role in allergic inflammation. Recent findings suggest that MIF is involved in several allergic disorders, including AD. In this study, MIF knockout (KO), MIF transgenic (Tg) and WT littermate mice were immunized with ragweed (RW) pollen or Japanese cedar (JC) pollen and challenged via eye drops. We observed that the numbers of conjunctiva- and eyelid-infiltrating eosinophils were significantly increased in RW and JC pollen-sensitized MIF Tg compared with WT mice or MIF KO mice. The mRNA expression levels of eotaxin, interleukin (IL)-5 and IL-13 were increased in pollen-sensitized eyelid skin sites of MIF Tg mice. An in vitro analysis revealed that high eotaxin expression was induced in dermal fibroblasts by MIF combined with stimulation of IL-4 or IL-13. This eotaxin expression was inhibited by the treatment with CD74 siRNA in fibroblasts. These findings indicate that MIF can induce eosinophil accumulation in the conjunctiva and eyelid dermis exposed to pollen. Therefore, targeted inhibition of MIF might result as a new option to control pollen-induced allergic conjunctivitis and pollen dermatitis.

## Introduction

Ragweed (RW) pollen is a clinically important airborne allergen in North America and is one of the major causes of allergic conjunctivitis. The development of allergic conjunctivitis is identified by typical and constitutive Ag sensitization, and patients suffer from several inflammatory symptoms, including itching, redness, lid swelling and chemosis. Additionally, environmental factors cause exacerbation of allergic dermatitis by penetrating barrier-disrupted skin. Percutaneous entry of environmental allergens through barrier-disrupted skin is strongly associated with the induction of Th2-dominant immunological responses, which resulted prominent infiltration of eosinophils in the skin as is seen in atopic dermatitis (AD) [1]. Pollen dermatitis is a recently identified disease characterized by itchy erythema of the skin during the Japanese cedar (JC) pollen season (February–April) [2]. It has been postulated that pollen dermatitis is triggered by the contact with cedar pollen Ag, i.e. airborne contact dermatitis, as skin symptoms characteristically appear on exposed areas, such as the face [3]. Indeed, in some patients with AD, which is characterized by impaired skin barrier function, JC pollen can preferentially cause seasonal exacerbation of dermatitis in exposed areas [4].

Macrophage migration inhibitory factor (MIF) was the first lymphokine reported to prevent the random migration of macrophages [5]. Since the molecular cloning of MIF cDNA [6], MIF has been re-evaluated as a pro-inflammatory cytokine and pituitary derived hormone that potentiates endotoxemia [7, 8]. MIF plays an important role in delayed-type hypersensitivity [9]. MIF is now recognized as a cytokine that exhibits a broad range of immune and inflammatory activities, including the induction of inflammatory cytokines, and regulation of macrophage and lymphocyte proliferation [10]. CD74 (also known as a MHC class II invariant chain) is a type II transmembrane protein that was reported to be part of the MIF receptor complex, along with its signaling component, CD44, and /or the chemokine receptors CXCR2 and CXCR4 [11–13].

MIF has been shown to have the potential to exacerbate human allergic and inflammatory diseases such as asthma [14] and acute respiratory distress syndrome [15]. We have also reported that there is excessive expression of MIF mRNA and MIF protein in inflammatory skin lesions and in the sera from AD patients [16, 17], and that the serum MIF levels decrease as the clinical features of this disease improve, suggesting that MIF plays a pivotal role in the inflammatory response in AD [18]. These studies raise the possibility that MIF is an important component of Th2-mediated immunopathology in general, and might therefore be relevant to chronic inflammatory allergic conditions.

In the present study, we used MIF knockout (KO), MIF transgenic (Tg), and wild-type (WT) C57BL/6 mice to assess the potential role of MIF in the pathogenesis of allergic conjunctivitis and pollen dermatitis sensitized by RW or JC pollen, and challenged mice via pollen-containing eye drops applied on the eye and the eyelid. We demonstrated that the number of conjunctiva and eyelid-infiltrating eosinophils was significantly increased in pollen-sensitized MIF Tg mice, whereas that in MIF KO mice was lower, compared with WT mice. We subsequently investigated the effects of MIF and CD74 siRNA on the eotaxin expression of dermal fibroblasts.

## Materials and Methods

### Materials

The following materials were obtained from commercial sources: RW pollen from Polyscience Inc (Warrington, PA, USA); Purified Sugi Basic Protein (Japanese Cedar Pollen Allergen) from Funakoshi (Tokyo, Japan); Alhydrogel 2% from InvivoGen (San Diego, CA,USA); Nichiban^TM^ tape from Nichiban (Tokyo, Japan); mouse eotaxin-specific enzyme-linked immunosorbent assay (ELISA) kit from Genzyme TECHNE (Cambridge, MT); Isogen RNA extraction kit from Nippon Gene (Tokyo, Japan); M-MLV reverse transcriptase from GIBCO (Grand Island, NY, USA); Taq DNA polymerase from Perkin-Elmer (Norwalk, CO, USA); nylon membranes from Schleicher & Schuell (Keene, NH); TransIT-TKO from TAKARA (Tokyo, Japan) and recombinant mouse IL-4 and recombinant mouse IL-13 from R&D systems (Minneapolis, MN). Recombinant rat MIF (this recombinant MIF cross reacts with that of mice) was expressed in Escherichia coli BL21/DE3 (Novagen, Madison, WI) and was purified as described previously [19]. All other chemicals were of analytical grade.

### Mice

The MIF-over-expressing transgenic (Tg) mice were established following cDNA microinjection. Their physical and biochemical characteristics, including body weight (30 g approx.), blood pressure and the serum cholesterol and blood sugar levels, were normal, as reported previously [20]. The transgene expression was regulated by a hybrid promoter composed of the cytomegalovirus (CMV) enhancer and β-actin/ β-globin promoter, as reported previously [21]. The strain of the original MIF-Tg mice was ICR, which was backcrossed with C57BL/6 for at least 10 generations. The Tg mice were maintained by heterozygous sibling mating. The skin from Tg mice showed anapproximately 10-fold higher level of MIF mRNA expression than those from WT mice [22]. The MIF-deficient knockout (KO) mice were established by targeted disruption of the MIF gene as described previously [23], using a mouse strain bred onto a C57BL/6 background. The experiments using mice, which is MIF Tg, MIF KO and wild-type (WT) mice were conducted according to the guidelines set by the University of Toyama Institutional Animal Care and Use Committee under an approved protocol. All experiments were performed on six to eight-week-old female mice (Five mice were used in each group).

### Sensitization and challenge

Sensitization-challenge experiments with RW or JC pollen were performed as described previously with some modifications [24]. In brief, RW pollen was emulsified in 0.125M NaOH (1 mg/ml) and mixed for 48 h by tube rotator. Mice (6–8 weeks old) were systemically s.c. sensitized to the RW pollen extract (10 μl) and Alhydrogel 2% (190 μl) or JC pollen solution (10 μl) and Alhydrogel 2% (190 μl) on day 0. After one week, the mice were immunized i.p. with the RW pollen extract (10 μl) and PBS (190 μl) or JC pollen solution (10 μl) and PBS (190 μl). In preparation for the barrier disruption of the eyelid and antigen challenge via eye drop, the eyelid hair was clipped before the first tape stripping. On days 11 and 13, tape stripping was performed carefully by applying and removing Nichiban^TM^ tape six times for each eyelid area. On days 14, 15 and 16, the RW pollen extract (5 μl /eye) or JC pollen solution (5 μl /eye) was used for challenge via eye drops. Control mice were subjected to the clipping, tape stripping and challenge without sensitization. The treated ocular tissue and surrounding skin were collected 24 hours after the challenge to evaluate the conjunctival eosinophil infiltration and mast cell infiltration. Each mouse was anesthetized with 10% nembutal (Hospira, Osaka, Japan) prior to procedure. In brief, all mice were injected intraperitoneally with pentobarbital (10%) at 10 mg/kg, including control mice, and we waited for five minutes before macroscopically observing the mice and excising skin sections from the dorsal surface, which were used for the immunohistochemical staining, RT-PCR, ELISA assay studies.

### Histological analysis

To assess the cellular infiltrates in the conjunctiva and the eyelid skin, tissues were removed intact and immediately fixed in 10% formalin and embedded in paraffin. Serial 6 μm thick vertical plane sections were subjected to hematoxylin and eosin (H&E) or Toluidine Blue staining. Eosinophils and mast cells were counted under a microscope at a magnification of X400 and expressed as the mean number of the cells in five random fields (one section per mouse, five mice per group).

### Reverse Transcription-PCR analysis

Total RNA was extracted from the conjunctiva and the eyelid skin. RNA reverse transcription was performed with M-MLV reverse transcriptase using random hexamer primers and subsequent amplification using Taq DNA polymerase. PCR was carried out for 34 to 40 cycles with denaturation at 95°C for 30 sec, annealing from 51 to 62°C for 30 sec and extension at 72°C for 30 sec using a thermal cycler (PE Applied Biosystems Gene Amp PCR system 9700, Life Technologies Japan, Tokyo, Japan). The mouse MIF primers used in the present study were 5’-gtttctgtcggagctcac-3’ (55–72) (forward) and 5’-agcgaaggtggaaccgttcca-3’ (215–236) (reverse). The eotaxin primers used were: 5’-ccaaggacttggcttcatgtag-3’ (438–459) (forward) and 5’-attctggcttggcatggtagc-3’ (912–932) (reverse). The IL-4 primers used were: 5’- acggcacagagctattgatg-3’ (71–90) (forward) and 5’- atggtggctcagtactacga-3’ (505–524) (reverse). The IL-5 primers used were: 5’- aggatgcttctgcacttga-3’(50–68) (forward), 5’- acaccaaggaactcttgca-3’(396–414) (reverse). The IL-13 primers used were: 5’- gacccagaggatattgcatg-3’ (320–339) (forward) and 5’- ccagcaaagtctgatgtgag-3’ (514–533) (reverse). The IFN-γ primers used were: 5’- gctctgagacaatgaacgct-3’ (98–117) (forward) and 5’-cgtctcggtctaatagagaaa-3’ (306–326) (reverse). GAPDH was used as a positive control. The primers used were: 5’-gaaggtcggtgtgaacggatttg-3’ (6–28) (forward) and 5’-gtccaccaccctgttgctgtagc-3’ (949–971) (reverse). After PCR, the amplified products were analyzed using 2% agarose gel electrophoresis.

### Cell culture, siRNA transfection and cytokine stimulation

Skin specimens were obtained from the dorsal surfaces of newborn MIF KO, WT and MIF Tg mice. The skin specimens were cut into 3 to 5 mm pieces and placed on a large petri dish with the subcutaneous side down, followed by tissue incubation for one week in a humidified atmosphere of 5% CO_2_ at 37°C. Once a sufficient number of fibroblasts had migrated out of the skin sections, the pieces of the skin were removed and the cells were passaged by trypsin digestion in the same manner as wound-harvested fibroblasts. Fibroblasts were grown in Dulbecco's modified Eagle's medium (DMEM) containing 10% FCS and 1% penicillin/streptomycin. After three passages, the fibroblasts were used for the experiments. CD74-specific siRNA was transfected at a concentration of 1 μM using TransIT-TKO according to the manufacturer's instructions. The MTT assay was performed after 24, 48 and 72 hours in order to assess the cell viability (data not shown). Forty-eight hours after the transfection, the cells were stimulated with IL-4 (10 ng ml^-1^), IL-13 (10 ng ml^-1^) and MIF (100 ng ml^-1^) alone or in combination for 24 hours. The cells were analyzed using reverse transcriptase-PCR. The culture supernatants were analyzed for eotaxin by ELISA.

### Statistical analysis

The values are expressed as the means ± SD of the respective test or control group. The statistical significance of differences between the control and test groups was evaluated by Student’s t-test.

## Results

### Effect of the tape stripping procedure on eosinophil infiltration in eyelid skin

**Figure 1:**
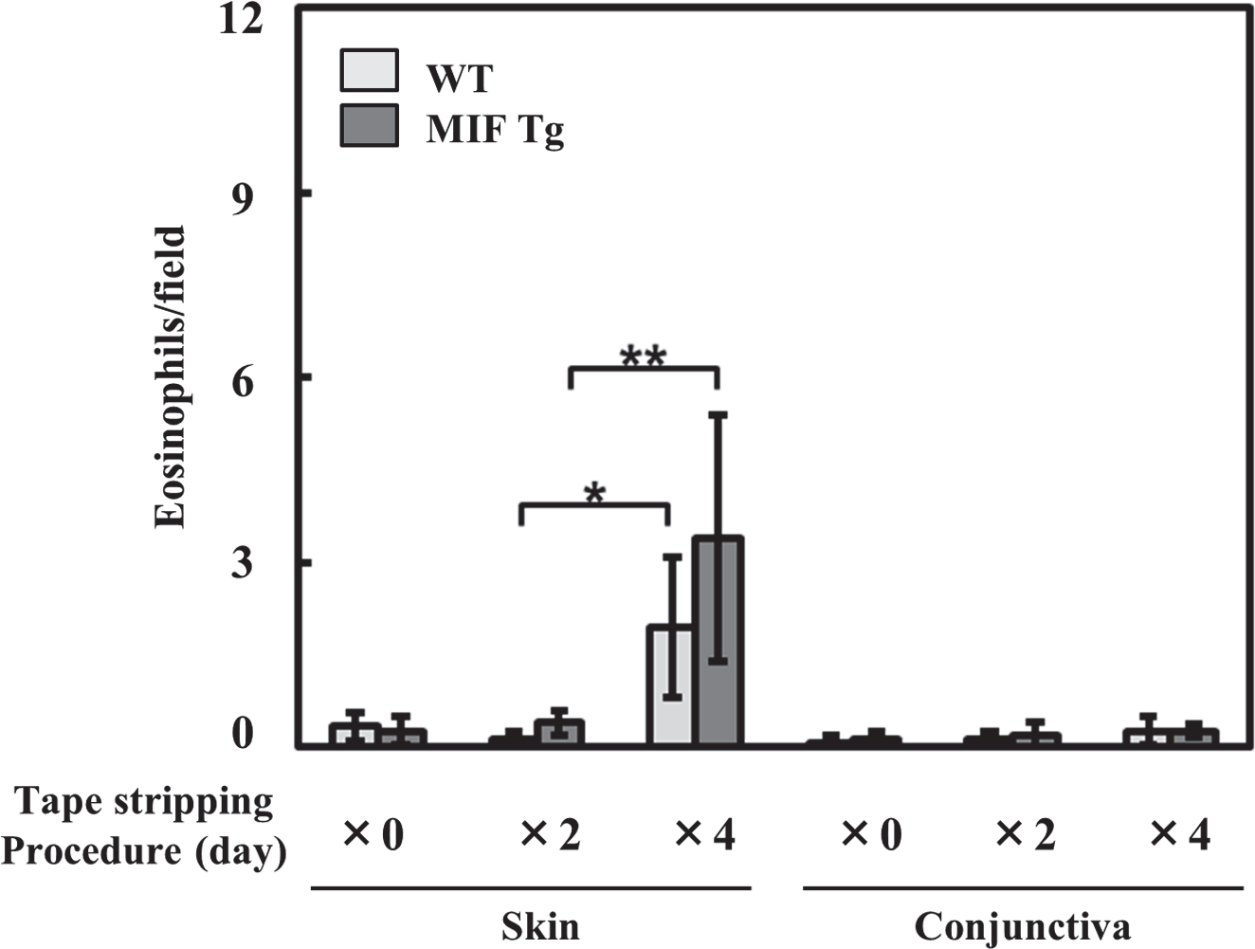
Effects of tape-stripping on the eosinophil infiltration in the eyelid skin. The hair was clipped before stripping on the eyelid skin sites of MIF Tg mice and WT mice. Then tape-stripping was performed every other day (X0: non stripping, X2: days 0 and days 2, X3: days 0, 2, 4, and 6). The application and removal of the adhesive tape was repeated six times per day. Twenty-four hours after final being tape-stripped, the skin was removed, and the eosinophil infiltration was evaluated by H&E staining. Each value represents the mean ± SD (n = 3, *P<0.05, **P<0.01). The Student's t-test was used.

We first evaluated the effect of tape stripping on eosinophil infiltration in eyelid skin. Four days stripping process induced eosinophil infiltration in the eyelid skin without sensitization (Fig. 1). To detect pollen-antigen specific and sensitive result, we determined the method that performes two days tape stripping.

### Induction of experimental pollen-induced AC and pollen dermatitis by active immunization

**Figure 2:**
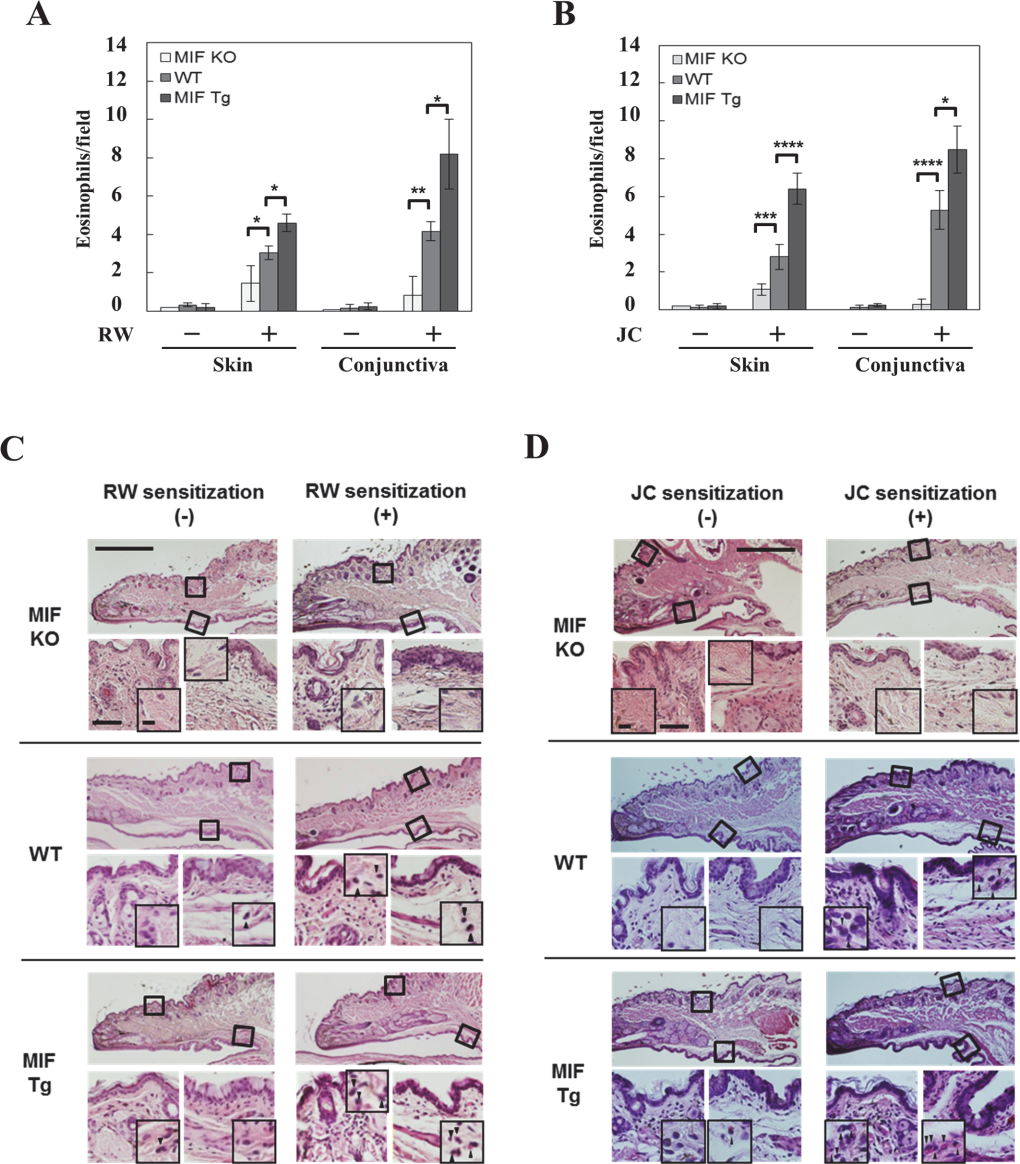
The eosinophil infiltration of experimental pollen-induced conjunctivitis and dermatitis following active immunization. The number of eosinophils in (A) RW and (B) JC pollen-sensitized conjunctiva and eyelid skin sites of MIF KO and MIF Tg mice were compared with those in WT mice. Each value represents the mean ± SD (n = 5 in each group, *P<0.05, **P<0.005, ***P<0.0005, ****P<0.0001). The Student's t-test was used. (C and D) The histological features of RW (C) and JC (D) pollen-sensitized conjunctiva and eyelid skin sites of MIF KO, MIF Tg and WT mice. HE-stained section of the palpebral part (upper panel), eyelid skin (lower left panel) and conjunctiva (lower right panel) are shown. The arrowheads point to eosinophils. The scale bars indicate 500 μm (upper panel), 50 μm (lower large panel) and 10 μm (lower small panel).

**Figure 3:**
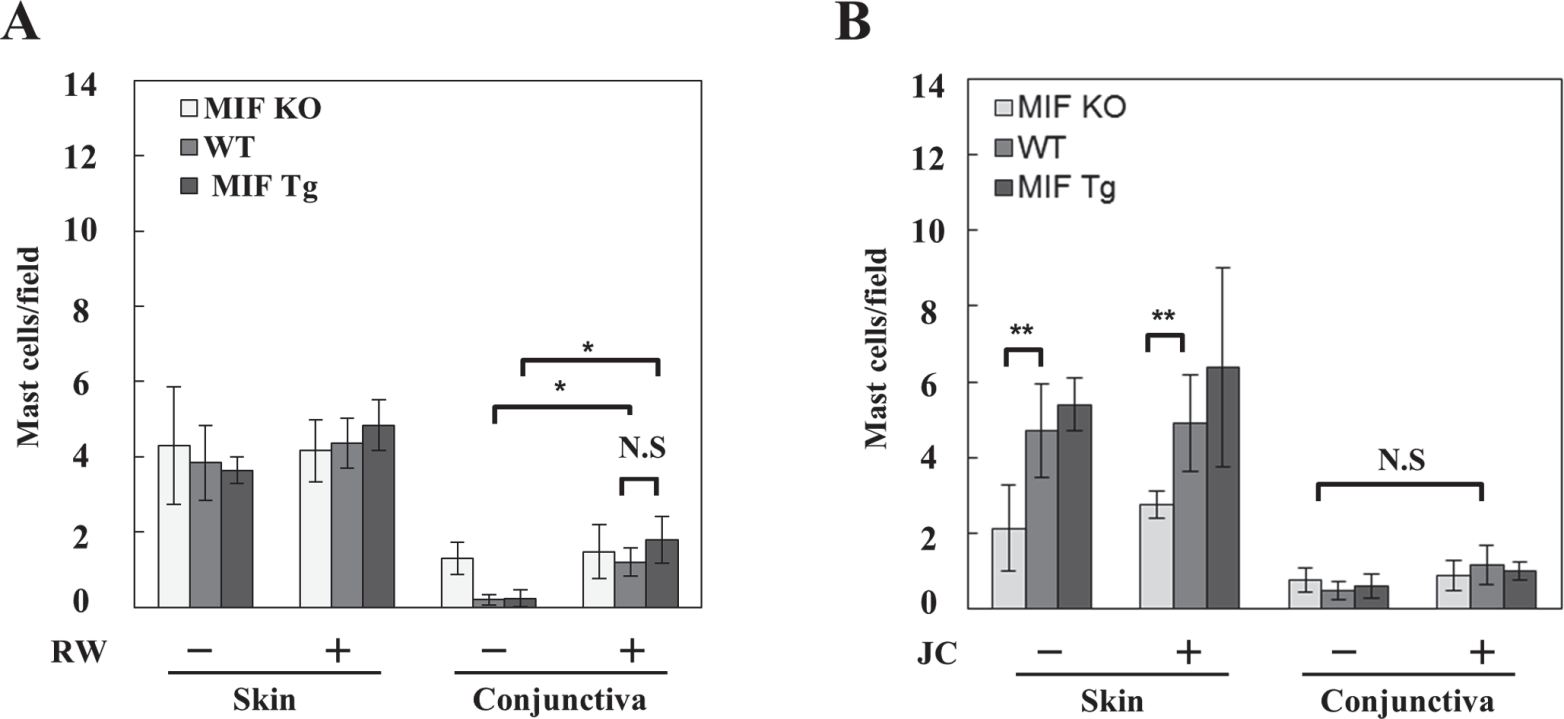
The number of mast cells in conjunctiva and eyelid skin site following active immunization by RW and JC pollen. The number of mast cells in (A) RW and (B) JC pollen-sensitized conjunctiva and eyelid skin sites of MIF KO and MIF Tg mice were compared with those in WT mice. Each value represents the mean ± SD (n = 5 in each group, *P<0.05, **P<0.01). The Student's t-test was used.

To examine the role of MIF in the eosinophilic infiltration in the conjunctiva and eyelid, MIF KO, MIF Tg and WT mice were immunized with RW or JC pollen and challenged via eye drops on the eye and the barrier-disrupted eyelid as described in the Materials and Methods section. The results showed that in both the conjunctiva and eyelid skin, only few eosinophils were present in non-sensitized control MIF KO, MIF Tg and WT mice, while eosinophilic infiltration of the conjunctival mucosa and the dermis was significantly increased following RW or JC sensitization. The mean number of eosinophils after RW pollen sensitization was 8.2 ± 1.82 (conjunctiva) and 4.6 ± 0.44 (eyelid skin) in MIF Tg mice, but only 4.2 ± 0.50 (conjunctiva) and 3.0 ± 0.34 (eyelid skin) in WT mice, 0.8 ± 0.99 (conjunctiva) and 1.4 ± 0.92 (eyelid skin) in MIF KO mice (*P<0.05, **P<0.005; Fig. 2A). The mean number of eosinophils after JC pollen sensitization was 8.5 ± 1.24 (conjunctiva) and 6.4 ± 0.82 (eyelid skin) in MIF Tg mice, but only 5.3 ± 1.01 (conjunctiva) and 2.9 ± 0.66 (eyelid skin) in WT mice, 0.3 ± 0.27 (conjunctiva), 1.1 ± 0.3 (eyelid skin) in MIF KO mice (*P<0.05, ***P<0.0001, ****P<0.0001; Fig. 2B). Fig. 2 shows the histological features of the RW (C) and JC (D) pollen-sensitized conjunctiva and skin sites in MIF KO, MIF Tg and WT mice. We then examined the mast cell infiltration in the conjunctiva and eyelid skin. The mast cell infiltration was increased following RW pollen-sensitization (P<0.05) in the conjunctiva. However, no significant differences were found among the RW pollen-sensitized MIF KO, MIF Tg and WT mice in either the conjunctiva or the eyelid skin (Fig. 3).

### The expression levels of eotaxin and Th2 type cytokines were increased in RW pollen-sensitized skin of MIF Tg mice

**Figure 4:**
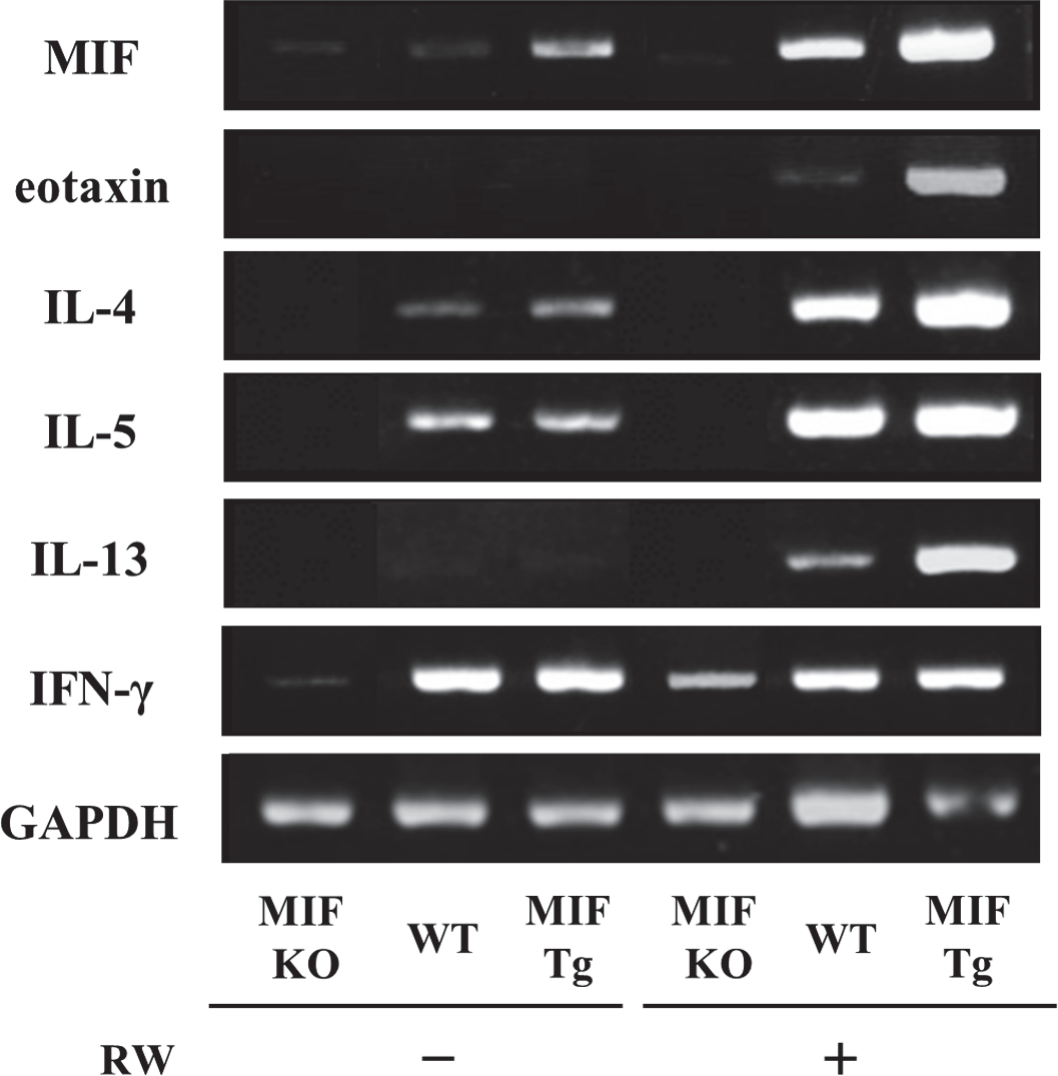
The expression of eotaxin and Th2-type cytokines in RW pollen-sensitized conjunctivitis and pollen dermatitis. The mRNA expression of eotaxin IL-4, IL-5, IL-13 and IFN-γ in RW pollen-sensitized eyelid skin (MIF KO, MIF Tg, and WT mice). The skin from MIF Tg mice showed approximately 10-fold higher level of MIF mRNA expression than those from WT mice GAPDH, gltceraldehyde-3-phosphate dehydrogenase. The data shown are representative out of three independent experiments.

We next examined the expression levels of mRNAs for eotaxin and cytokines in RW pollen-sensitized eyelid skin tissue samples from both MIF Tg and WT mice.The expression levels of eotaxin and Th2 type cytokines were increased in the RW pollen-sensitized skin of MIF Tg mice compared with that of WT mice. Conversely, low mRNA expression of eotaxin, IL-4, IL-5, and IL-13 was observed in pollen-sensitizes eyelid skin sites of MIF KO mice (Fig. 4). However, the level of IFN-γ did not differ between the MIF Tg and WT mice.

### CD74 siRNA transfection inhibits MIF-induced eotaxin expression and production by cultures fibroblasts from WT mice

**Figure 5:**
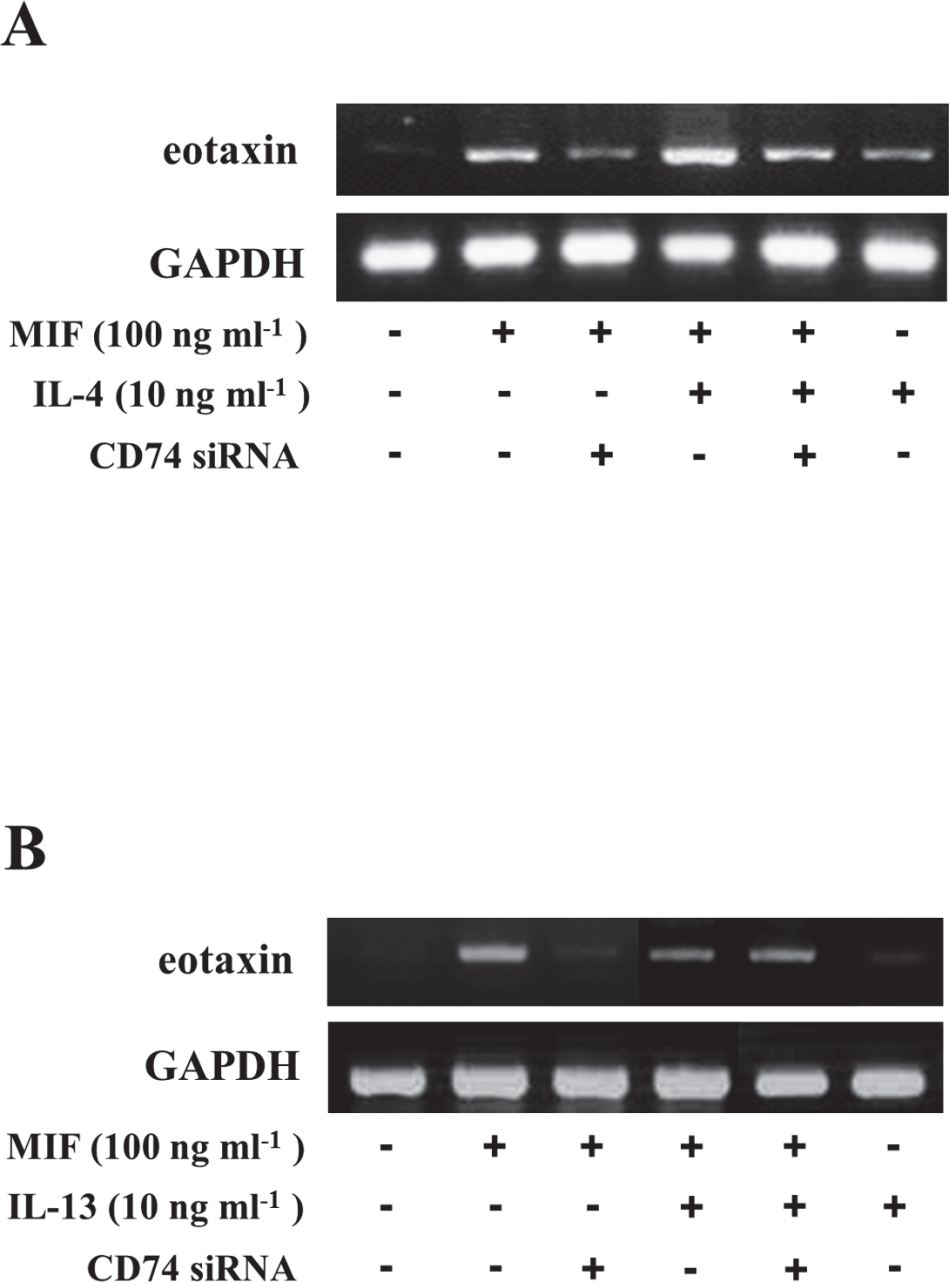
CD74 siRNA transfection inhibits MIF-induced eotaxin mRNA expression by cultured fibroblasts from WT mice. CD74-specific siRNA was transfected into cultured mouse fibroblasts at a concentration of 1 μM. Forty-eight hours after transfection, the cells were stimulated with (A) IL-4 (10 ng ml^-1^), (B) IL-13 (10 ng ml^-1^) and MIF (100 ng ml^-1^) alone or in combination for 24 hours. The cells were harvested by the addition of lysis reagents. RNA was extracted from the cells, and the abundance of eotaxin mRNA was evaluated by reverse transcriptase-PCR. The data are from a representative experiment that was repeated three times and yielded similar results.

**Figure 6:**
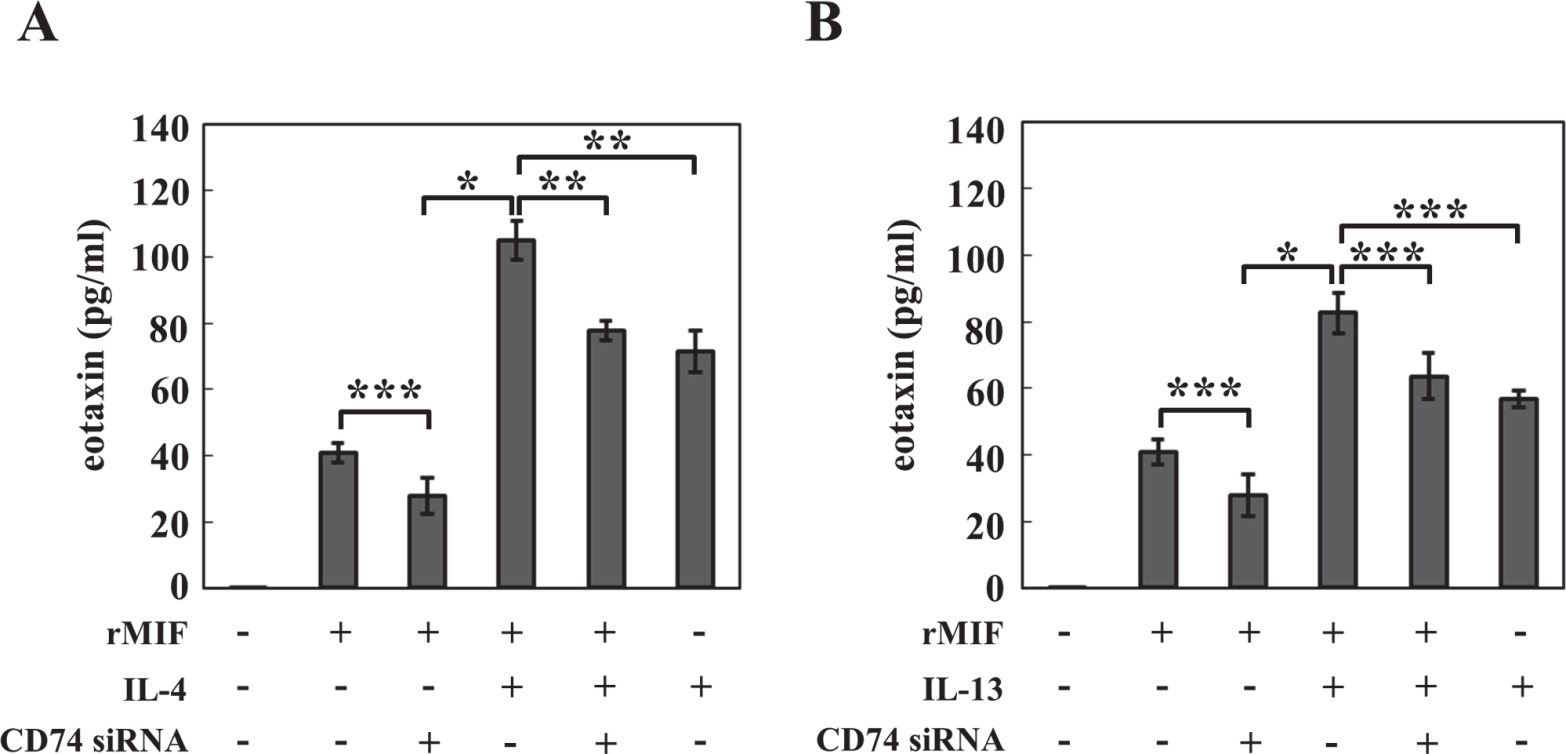
CD74 siRNA transfection inhibits MIF-induced eotaxin protein production by cultured fibroblasts from WT mice. CD74-specific siRNA was transfected into cultured mouse fibroblasts at a concentration of 1 μM. Forty-eight hours after transfection, the cells were stimulated with (A) IL-4 (10 ng ml^-1^), (B) IL-13 (10 ng ml^-1^) and MIF (100 ng ml^-1^) alone or in combination for 24 hours. The eotaxin content of the culture supernatants was analyzed by ELISA. The data are from a representative experiment that was repeated three times and yielded similar results. Each value represents the mean ±SD of three specimens. *P<0.001, **P<0.01, ***P<0.05. The Student's t-test was used.

Eotaxin has been described as a key mediator of the development of tissue eosinophilia. In dermal fibroblasts from WT mice, single stimulation with IL-4, IL-13 or MIF significantly induced the expression of eotaxin mRNA and the production of eotaxin protein alone compared to unstimulated fibroblasts. Combined stimulation with MIF and IL-4 or with MIF and IL-13 further increased the eotaxin secretion (Figs. 5 and 6). Further, fibroblasts from MIF KO, WT and MIF Tg mice were stimulated with recombinant IL-4 and IL-13 alone and in combination with CD74 siRNA. The IL-4 and IL-13 stimulation caused increased eotaxin production in fibroblasts from WT mice compared to fibroblasts from MIF KO mice (P<0.01 MIF KO vs. WT and P<0.01 WT vs. MIF KO). Further, combined treatment with CD74 siRNA resulted in reduced production of eotaxin in fibroblasts from WT mice compared to treatment with the cytokines alone (P<0.01 vs. IL-4 alone WT and P<0.01 vs. IL-13 alone WT).

Moreover, a significant increase in the eotaxin production was observed in MIF Tg mice following stimulation with IL-4 and IL-13 compared to the MIF KO and WT mice. However, in the case of combination treatment with CD74 siRNA, decreased eotaxin production was observed compared to that occurring after treatment with IL-4 and IL-13 alone in the MIF Tg mice (Table 1).

**Table 1:** Effects of CD74 siRNA transfection on MIF, IL-4 and IL-13-induced eotaxin production.

	MIF KO	WT	MIF Tg
－	N.D	0.9 ± 1.0	6.6 ± 3.7
IL-4 (10 ng/ml)	2.6 ± 1. 9 ^a^	22.3 ± 4.7 ^b^	59.5 ± 2.5 ^a^ ^,^ ^b^
CD74 siRNA +IL-4	－	18.2 ± 3.2 ^c^	44.5 ± 6.5 ^a^ ^.^ ^c^
IL-13 (10 ng/ml)	1.7 ± 0.7 ^a^	19.3 ± 1.8 ^b^	46.8 ± 4.8 ^a^ ^,^ ^b^
CD74 siRNA +IL-13	－	15.6 ± 2.4 ^d^	31.7 ± 3.4 ^a^ ^.^ ^d^

The data are from a representative experiment that was repeated three times and yielded similar results. Each value represents the mean ±SD of three specimens.

^**a**^ vs WT, *P*<0.01,

^**b**^ vs MIF KO, *P*<0.01,

^**c**^ vs IL-4, *P*<0.01,

^**d**^ vs IL-13, *P*<0.01.

The mechanism(s) involved in the receptor-mediated MIF-induced eotaxin secretion, especially MIF-mediated IL-4 and MIF-mediated IL-13-induced eotaxin expression, are unclear. We performed *in vitro* experiments to clarify the role of the MIF receptor in eotaxin expression. Transfection of CD74 siRNA 48 hours before MIF stimulation significantly inhibited the MIF-induced eotaxin expression (Fig. 5) and production (***P<0.05; Fig. 6A, B), and the combined stimulation (MIF and IL-4 or MIF and IL-13)-induced eotaxin expression (Fig. 3) and production (MIF and IL-4 [**P<0.01; Fig. 6A], or MIF and IL-13 [***P<0.05; Fig. 6B]). These data suggest that CD74 has a pivotal role in MIF-induced eotaxin expression in mouse dermal fibroblasts.

## Discussion

Allergic conjunctivitis is one of the most common symptoms of pollinosis, and is characterized by a spectrum of disorders ranging from acute forms of seasonal conjunctivitis to chronic and severe sight-threatening forms, such as vernal kerato conjunctivitis. Additionally, patients with AD have a baseline-impaired barrier function that allows exogenous proteins, microbes and other irritants easier access into the epidermis, unlike a normally functioning epidermis that prevents the penetration beyond the stratum corneum.

In this study, we established a mouse model of RW and JC pollen-specific conjunctivitis and eyelid dermatitis in skin barrier-disrupted mice. We have modified the methods of Magone *et al*. and induced RW or JC- pollen specific conjunctivitis and eyelid dermatitis, with repeated tape-stripping around the eyes before the RW or JC pollen challenge, which resulted in increased eosinophil infiltration following RW or JC pollen sensitization/challenge in eyelid skin sites, as well as in the conjunctiva.

In the recent literature, the prevalence of pollinosis in childhood has been increasing [25], especially in children bearing filaggrin mutations [26]. Indeed, seasonal flares may also be relevant, as one case series described a patient with severe eczema only during the spring and fall with positive skin-prick and patch test to RW pollen [27]. These reports suggest that the presence of skin barrier dysfunction initiates the penetration of pollen allergens, and that pollen is responsible for the aggravation of AD. Kusunoki *et al*. also demonstrated that, among children with AD, there was a statistically significant correlation between the severity of AD and the presence of Japaneses cedar pollinosis, and children with cedar pollinosis tended to have more severe AD symptoms [28]. Furthermore, in patients with JC pollen dermatitis, patch testing with cedar pollen Ag to mechanically injured skin, such as that following scratching or tape stripping, induces a positive reaction after 48 h [4].

Our findings demonstrate that the number of conjunctiva and eyelid-infiltrating eosinophils was significantly increased in pollen-sensitized MIF Tg mice compared to WT mice. We recently reported that MIF induces eosinophil infiltration in the skin [22]. The number of skin-infiltrating eosinophils was also significantly increased in OVA-sensitized MIF Tg mice. In the present study, we have also shown that the expression of both eotaxin and IL-5 is markedly increased in the pollen-sensitized conjunctiva and eyelid skin of MIF Tg mice. The other Th2-type cytokines, IL-4 and IL-13, were also slightly increased in MIF Tg mice. We have previously demonstrated that MIF increases the eosinophil infiltration into the OVA-sensitized skin and enhances the expression of eotaxin and Th2-type cytokines, especially IL-5, from cultured fibroblasts [22]. These results are in accordance with the findings of a previous report that demonstrated that MIF plays an important role in IL-5-induced eosinophilopoiesis and tissue eosinophilia [29]. High levels of MIF were also detected in the lacrimal fluid of patients with severe AD [30]. It was demonstrated, the tear MIF concentration was significantly higher in patients with severe AD and patients with allergic conjunctivitis than in healthy controls. These studies raise the possibility that MIF is an important component of Th2-mediated immunopathology. The accumulation of eosinophils is characteristic of the inflammation associated with AD and allergic conjunctivitis. Taken together, previous observations and our current data indicate that MIF significantly contributes to tissue eosinophilia in allergic inflammation, which initiates local inflammatory responses and sustains chronic inflammation.

MIF is an intracellular protein that can be released into the extracellular environment where it acts as a potent inflammatory stimulant and contributes significantly to the innate and adaptive immune responses. However, the mechanisms of cellular binding and activation remain to be fully clarified. The MIF receptor, CD74, is widely expressed on different cell types. Several studies have reported that CD74 is expressed on both the cell surface and intra-cellularly in B cell lymphoma [31], T cell lymphoma, melanoma cells [32] and gastric epithelial cells [33]. Although the extracellular domain of CD74 has been shown to bind MIF with high affinity, recent reports showed that the ligand- receptor interaction can occur either on the cell surface or within an endosome [34, 35].

We have previously reported that MIF has the ability to increase the secretion of eotaxin from cultured fibroblasts. Combined stimulation with IL-4 also stimulated the eotaxin production. To clarify the role of receptor-mediated MIF signaling pathways in eotaxin expression, we examined the contribution of CD74 on mouse skin fibroblasts. We have demonstrated that the transfection of CD74 siRNA 48 hours before MIF stimulation significantly inhibited the MIF-induced eotaxin expression and production, and that combined stimulation (MIF and IL-4 or MIF and IL-13) induced eotaxin expression and production. Recently, it was demonstrated that MIF’s functions are dependent on the activation of its receptor, CD74, which acts in concert with eotoxin in a positive-feedback loop to activate eosinophils in allergic conditions [36].

These results suggested that skin fibroblasts could respond to extracellular MIF, and indicated that a CD74-mediated signal transduction mechanism was involved in the MIF-mediated IL-4 (or IL-13)-induced eotaxin release. Therefore, MIF and its receptor, CD74, may be useful targets to reduce eosinophilic skin inflammation.

AD and pollinosis are common chronic allergic diseases associated with the activation of T-helper 2 cells. It has recently been reported that twin cases of AD showed similar clinical manifestations, including aggravation of facial skin lesions during the cedar pollen season, followed by severe scratching behavior resulting in generalization of eczema [37]. AD patients can be sensitized more easily, and eczematous lesions can be quickly caused by attached allergens. Airborne JC pollen has also been increasing annually, although yearly variations are seen, thus leading to a marked increase in the number of patients with pollinosis characterized by seasonal rhinoconjunctivitis due to an allergy to cedar pollen [38]. Therefore, the targeted inhibition of MIF might result as a new option to control pollen-induced allergic conjunctivitis and pollen dermatitis.

## References

[1] Kondo H, Ichikawa Y, Imokawa G (1998) Percutaneous sensitization with allergens through barrier-disrupted skin elicits a Th2-dominant cytokine response. Eur J Immunol 28: 769–779. 9541570 10.1002/(SICI)1521-4141(199803)28:03<769::AID-IMMU769>3.0.CO;2-H

[2] Yokozeki H, Satoh T, Katayama I, Nishioka K (2007) Airborne contact dermatitis due to Japanese cedar pollen. Contact Dermatitis 56: 224–228. 17343624 10.1111/j.1600-0536.2004.00491.x

[3] Oiwa M, Satoh T, Watanabe M, Niwa H, Hirai H, et al (2008) CRTH2-dependent, STAT6-independent induction of cedar pollen dermatitis. Clin Exp Allergy 38: 1357–1366. 10.1111/j.1365-2222.2008.03007.x 18477017

[4] Yokozeki H, Takayama K, Katayama I, Nishioka K (2006) Japanese cedar pollen as an exacerbation factor in atopic dermatitis: results of atopy patch testing and histological examination. Acta Derm Venereol 86: 148–151. 16648919 10.2340/00015555-0020

[5] Bloom BR, Bennett B (1966) Mechanism of reaction in vivo associated with delayed-type hypersensitivity. Science 153: 80–82. 5938421 10.1126/science.153.3731.80

[6] Weiser WY, Temple PA, Witek-Giannotti JS, Remold HG, Clark SC, et al (1989) Molecular cloning of a cDNA encoding a human macrophage migration inhibitory factor. Proc Natl Acad Sci USA 86: 7522–7526. 2552447 10.1073/pnas.86.19.7522 PMC298097

[7] Bucala R (1996) MIF re-evaluated: pituitary hormone and glucocorticoid-induced regulator of cytokine production. FASEB J 7: 19–24. 10.1016/1359-6101(96)00008-1 8864351

[8] Bernhagen J, Calandra T, Mitchell RA, Martin SB, Tracey KJ, et al (1993) MIF is a pituitary-derived cytokine that potentiates lethal endotoxaemia. Nature 365: 756–759. 8413654 10.1038/365756a0

[9] Bernhagen J, Calandra T, Bucala R (1998) Regulation of the immune response by macrophage migration inhibitory factor: biological and structural features. J Mol Med 76: 151–161. 9535548 10.1007/s001090050204

[10] Das R, Moss JE, Robinson E, Roberts S, Levy R, et al (2011) Role of macrophage migration inhibitory factor in the Th2 immune response to epicutaneous sensitization. J Clin Immunol 31: 666–680. 10.1007/s10875-011-9541-7 21559932 PMC3700537

[11] Leung DY, Nicklas RA, Li JT, Bernstein IL, Boguniewicz M, et al (2004) Disease management of atopic dermatitis: an updated practice parameter. Joint Task Force on Practice Parameters. Ann Allergy Asthma Immunol 93: S1–21. 15478395 10.1016/s1081-1206(10)61385-3

[12] Schwartz V, Krüttgen A, Weis J, Weber C, Ostendorf T, et al (2012) Role for CD74 and CXCR4 in clathrin-dependent endocytosis of the cytokine MIF. Eur J Cell Biol 91: 435–449. 10.1016/j.ejcb.2011.08.006 22014447

[13] Benedek G, Meza-Romero R, Andrew S, Leng L, Burrows GG, et al (2013) Partial MHC class II constructs inhibit MIF/CD74 binding and downstream effects. Eur J Immunol 43: 1309–1321. 10.1002/eji.201243162 23576302 PMC3788583

[14] Rossi AG, Haslett C, Hirani N, Greening AP, Rahman I, et al (1998) Human circulating eosinophils secrete macrophage migration inhibitory factor (MIF): potential role in asthma. J Clin Invest 15: 2869–2874. 10.1172/JCI1524 PMC508878 9637721

[15] Donnelly SC, Haslett C, Reid PT, Grant IS, Wallace WA, et al (1997) Regulatory role for macrophage migration Inhibitory factor in acute respiratory distress syndrome. Nature Med 3: 320–323. 9055860 10.1038/nm0397-320

[16] Shimizu T, Abe R, Ohkawara A, Nishihira J (1999) Increased production of macrophage migration inhibitory factor (MIF) by PBMCs of atopic dermatitis. J Allergy Clin Immunol 104: 659–664. 10482843 10.1016/s0091-6749(99)70339-8

[17] Shimizu T (2005) Role of macrophage migration inhibitory factor (MIF) in the skin. J Dermatol Sci 37: 65–73. 15659324 10.1016/j.jdermsci.2004.08.007

[18] Shimizu T, Abe R, Ohkawara A, Mizue Y, Nishihira J (1997) Macrophage migration inhibitory factor is an essential immunoregulatory cytokine in atopic dermatitis. Biochem Biophys Res Commun 240: 173–178. 9367905 10.1006/bbrc.1997.7633

[19] Shimizu T, Nishihira J, Watanabe H, Abe R, Ishibashi T, et al (2004) Cetirizine, an H1-receptor antagonist, suppresses the expression of macrophage migration inhibitory factor (MIF): Its potential anti-inflammatory action. Clin Exp Allergy 34: 103–109. 14720269 10.1111/j.1365-2222.2004.01836.x

[20] Sasaki S, Nishihira J, Ishibashi T, Yamasaki Y, Obikane K, et al (2004) Transgene of MIF induces podocyte injury and progressive mesangial sclerosis in the mouse kidney. Kidney Int 65: 469–481. 14717917 10.1111/j.1523-1755.2004.00394.x

[21] Akagi Y, Isaka Y, Akagi A, Ikawa M, Takenaka M, et al (1997) Transcriptional activation of a hybrid promoter composed of cytomegalovirus enhancer and beta-actin/beta-globin gene in glomerular epithelial cells in vivo. Kidney Int 51: 1265–1269. 9083295 10.1038/ki.1997.172

[22] Yoshihisa Y, Makino T, Matsunaga K, Honda A, Norisugi O, et al (2011) Macrophage migration inhibitory factor is essential for eosinophil recruitment in allergen-induced skin inflammation. J Invest Dermatol 131: 925–931. 10.1038/jid.2010.418 21191413

[23] Honma N, Koseki H, Akasaka T, Nakayama T, Taniguchi M, et al (2000) Deficiency of the macrophage migration inhibitory factor gene has no significant effect on endotoxaemia. Immunology 100: 84–90. 10809963 10.1046/j.1365-2567.2000.00011.x PMC2326981

[24] Schopf L, Luccioli S, Bundoc V, Justice P, Chan CC, et al (2005) Differential modulation of allergic eye disease by chronic and acute ascaris infection. Invest Ophthalmol Vis Sci 46: 2772–2780. 16043850 10.1167/iovs.04-0899

[25] Grize L, Gassner M, Wüthrich B, Bringolf-Isler B, Takken-Sahli K, et al (2006) Swiss Surveillance Programme on Childhood Allergy and Respiratory symptoms with respect to Air Pollution (SCARPOL) team. Trends in prevalence of asthma, allergic rhinitis and atopic dermatitis in 5–7-year old Swiss children from 1992 to 2001. Allergy 61: 556–562. 16629784 10.1111/j.1398-9995.2006.01030.x

[26] Weidinger S, O'Sullivan M, Illig T, Baurecht H, Depner M, et al (2008) Filaggrin mutations, atopic eczema, hay fever, and asthma in children. J Allergy Clin Immunol 121: 1203–1209. 10.1016/j.jaci.2008.02.014 18396323

[27] Adinoff AD, Tellez P, Clark RA (1988) Atopic dermatitis and aeroallergen contact sensitivity. J Allergy Clin Immunol 81: 736–742. 3356851 10.1016/0091-6749(88)91047-0

[28] Kusunoki T, Korematsu S, Nakahata T, Hosoi S (2002) Cedar pollinosis in Japanese schoolchildren: results from a large questionnaire-based survey. Arerugi 51: 15–19. 11877958

[29] Magalhães ES, Paiva CN, Souza HS, Pyrrho AS, Mourão-Sá D, et al (2009) Macrophage migration inhibitory factor is critical to interleukin-5-driven eosinophilopoiesis and tissue eosinophilia triggered by Schistosoma mansoni infection. FASEB J 23: 1262–1271. 10.1096/fj.08-124248 19088181

[30] Kitaichi N, Shimizu T, Honda A, Abe R, Ohgami K, et al (2006) Increase in macrophage migration inhibitory factor levels in lacrimal fluid of patients with severe atopic dermatitis. Graefes Arch Clin Exp Ophthalmol 244: 825–828. 16331484 10.1007/s00417-005-0168-3

[31] Wraight CJ, van Endert P, Möller P, Lipp J, Ling NR, et al (1990) Human major histocompatibility complex class II invariant chain is expressed on the cell surface. J Biol Chem 265: 5787–5792. 1690714

[32] Ong GL, Goldenberg DM, Hansen HJ, Mattes MJ (1999) Cell surface expression and metabolism of major histocompatibility complex class II invariant chain (CD74) by diverse cell lines. Immunology 98: 296–302. 10540230 10.1046/j.1365-2567.1999.00868.x PMC2326920

[33] Barrera CA, Beswick EJ, Sierra JC, Bland D, Espejo R, et al (2005) Polarized expression of CD74 by gastric epithelial cells. J Histochem Cytochem 53: 1481–1489. 15923369 10.1369/jhc.4A6552.2005 PMC3957538

[34] Lue H, Dewor M, Leng L, Bucala R, Bernhagen J (2011) Activation of the JNK signalling pathway by macrophage migration inhibitory factor (MIF) and dependence on CXCR4 and CD74. Cell Signal 23: 135–144. 10.1016/j.cellsig.2010.08.013 20807568 PMC3586206

[35] Xie L, Qiao X, Wu Y, Tang J (2011) β-Arrestin1 mediates the endocytosis and functions of macrophage migration inhibitory factor. PLoS One 6: e16428 10.1371/journal.pone.0016428 21283538 PMC3026819

[36] Calheiros AS, Mesquita-Santos FP, Magalhães ES, Mourão-Sá D, et al (2011) Cross-talk between macrophage migration inhibitory factor and eotaxin in allergic eosinophil activation forms leukotriene C₄-synthesizing lipid bodies. Am J Respir Cell Mol Biol 44: 509–516. 10.1165/rcmb.2010-0004OC 20539011

[37] Murakami Y, Matsui S, Kijima A, Kitaba S, Murota H, et al (2011) Cedar pollen aggravates atopic dermatitis in childhood monozygotic twin patients with allergic rhino conjunctivitis. Allergol Int 60: 397–400. 10.2332/allergolint.10-CR-0268 21430436

[38] Tanihara S, Oki I, Ojima T, Nakamura Y, Yanagawa H (1999) Process and current status of the epidemiologic studies on cedar pollinosis in Japan. J Epidemiol 9: 20–26. 10098349 10.2188/jea.9.20

