# Correction: Role of Macrophage Migration Inhibitory Factor (MIF) in Pollen-Induced Allergic Conjunctivitis and Pollen Dermatitis in Mice

**DOI:** 10.1371/journal.pone.0122546

**Published:** 2015-03-19

**Authors:** The PLOS ONE Staff

The fifth author’s name is spelled incorrectly. The correct name is: Nobuyoshi Kitaichi.

The correct citation is: Nagata Y, Yoshihisa Y, Matsunaga K, Rehman MU, Kitaichi N, Shimizu T. (2015) Role of Macrophage Migration Inhibitory Factor (MIF) in Pollen-Induced Allergic Conjunctivitis and Pollen Dermatitis in Mice. PLoS ONE 10(2): e0115593. doi:10.1371/journal.pone.0115593.
